# Epidemiological characterization and phylogenetic analysis of human metapneumovirus isolated from children in Ningbo, China, 2020–2024

**DOI:** 10.1099/mgen.0.001760

**Published:** 2026-06-22

**Authors:** Yongdong Li, Rui Liu, Lei Xie, Tianjie Wang, Hongxia Ni, Tianchi Yang, Xu Guo

**Affiliations:** 1 Municipal Key Laboratory of Virus Research, Ningbo Municipal Center for Disease Control and Prevention, Ningbo, PR China; 2 Huangpu Customs Technology Center, Guangzhou, PR China; 3 College of Veterinary Science, Anhui Agricultural University, Hefei, PR China

**Keywords:** children, epidemiology, human metapneumovirus, Ningbo, phylogenetic analysis

## Abstract

Human metapneumovirus (hMPV) is an important cause of acute respiratory infections in children, but genomic surveillance data from Ningbo, a coastal port city of China, remain limited. We investigated the epidemiology, genetic diversity and phylogeographic patterns of hMPV among children in Ningbo from 2020 to 2024 using 6,632 respiratory specimens from paediatric outpatients and 26 hMPV-positive samples. The overall hMPV-positive rate was 3.62% (240/6,632), with a peak in 2022 (6.86%). An atypical summer peak in 2022 and a prolonged 2023–2024 season suggested altered seasonality in the post-COVID-19 period. Preschool-aged children (1–6 years) were the most affected age group and the proportion of viral co-infections among hMPV-positive cases increased significantly over time. Phylogenetic analysis of the 26 genomes showed co-circulation of four lineages, with B2 and A2.2.2 predominating across multiple years. We identified one A2.2.2 strain (20240959) carrying a 111-nt (37-aa) insertion in the *G* gene, whereas other A2.2.2 strains retained the classical non-duplicated *G* sequence. Several lineage-associated substitutions in *F* protein were observed between lineages A, B1 and B2, and mapping suggested that a substantial proportion of these sites fell within predicted linear B-cell epitope-prone regions. Phylogeographic reconstruction indicated multiple introductions of hMPV into Ningbo from other parts of China and from overseas. These findings demonstrate the genetic and epidemiological complexity of hMPV circulation in a major port city and underscore the need for continued, genome-informed surveillance to monitor hMPV evolution.

**Impact Statement**

Based on 6,632 paediatric outpatient respiratory specimens collected in Ningbo (2020–2024), this study provides multi-year post-pandemic evidence on hMPV in a coastal port city, with an overall positive rate of 3.62% and a marked increase to 6.86% in 2022, alongside seasonal reshaping (an atypical summer peak in 2022 and a prolonged season in 2023–2024). Whole-genome sequencing of 26 viruses showed co-circulation of four lineages (A2.2.1, A2.2.2, B1 and B2) and identified an A2.2.2 strain (20240959) carrying a 111-nt (37-aa) insertion in the *G* gene, adding regional evidence relevant to genotype and potential antigenic implications. The findings are actionable for paediatric surveillance: infections clustered in children aged 1–6 years and co-infections increased from 6.1% (2020) to 32.6% (2024), supporting strengthened multiplex testing and targeted management. Phylogeographic analyses suggest multiple domestic and international introductions and the NCBI-deposited sequences provide a baseline for ongoing genomics-driven monitoring and early warning.

## Data Summary

All sequence data from this study are stored in the National Center for Biotechnology Information (NCBI). The accession numbers for the amplified sequences in this study are: PV252070 - PV252075 and PX608635 - PX608654. Reference sequence information can be found in Supplementary information 1.

## Introduction

Human metapneumovirus (hMPV), a member of the *Pneumoviridae* family, is a major respiratory pathogen that causes acute respiratory infections, particularly in young children, the elderly and immunocompromised individuals [1]. In children, hMPV is a major cause of bronchiolitis and pneumonia, accounting for ~10% of hospitalizations for acute respiratory tract infections. A proportion of infants and immunocompromised patients may require admission to the intensive care unit. Moreover, hMPV can co-infect with respiratory syncytial virus (RSV) [2,3], which further increases the risk of severe disease. Despite its clinical significance, genomic surveillance among children in China remains limited, particularly in port hubs like Ningbo, where international travel may contribute to viral diversity.

The hMPV is classified into two major genetic lineages, A and B, which are further subdivided into six sublineages: A1, A2.1, A2.2.1 (A2B1), A2.2.2 (A2b2/A2c), B1 and B2 [4]. The hMPV genome comprises eight annotated genes: *N*, *P*, *M*, *F*, *M2*, *SH*, *G* and *L*. Among all sublineages, the *N* gene is the most conserved, whereas the *G* gene is the least conserved [5]. Molecular epidemiology studies indicate that these lineages co-circulate within and between regions, with the predominant lineage often changing from year to year [1]. Large-scale phylodynamic analyses suggest that some earlier lineages, such as A1 and ancestral A2 variants, have declined or may be close to extinction [4,6], whereas A2.2.1 and especially A2.2.2 have expanded and now account for a substantial proportion of globally circulating strains [5,7, 8].

Ningbo, a coastal port city in eastern China, serves as a major transportation hub with extensive domestic and international travel, which may pose significant risks for the import and export of epidemic diseases. However, genomic surveillance of hMPV in this region has been limited, and little is known about the molecular epidemiology of hMPV circulating among children in Ningbo. In this study, we conducted a comprehensive epidemiological and phylogenetic analysis of hMPV strains detected in paediatric patients from 2020 to 2024. We aimed to characterize the seasonal and demographic patterns of hMPV infections, assess the genetic diversity of circulating strains through whole-genome sequencing, which allows for a comprehensive understanding of viral evolution compared to partial gene sequencing, and reconstruct the phylogeographic transmission dynamics of hMPV in Ningbo. These findings offer valuable insights into hMPV epidemiology and evolution, contributing to improved surveillance and public health preparedness.

## Methods

### Ethical approval and consent to participate

This work was approved by the Ningbo Municipal Center for Disease Control and Prevention Biomedical Research Ethics Review Committee (No.202105), which waived the requirement for informed consent to participate. All specimens were anonymized before analysis by assigning unique coded identifiers. The investigators had access only to coded samples and did not have access to directly identifiable patient information.

### Sample collection and detection

To investigate the epidemiological status of hMPV, nasopharyngeal swabs were collected from paediatric patients (under 18 years old) presenting influenza-like symptoms (axillary temperature ≥38 °C, either a sore throat or a cough), at the Fever Clinic of Women and Children’s Hospital of Ningbo University, Ningbo, China, between 2020 and 2024. Only samples from outpatient children were included in the study. Hospitalized patients were excluded, and samples of poor quality or improper collection (insufficient specimen volume, leakage, obvious contamination, incomplete or inconsistent labelling, or failure to meet the required collection, storage, or transport conditions) were also removed. To allow a more comprehensive investigation of hMPV in paediatric patients, we did not exclude samples from individuals with known or unknown underlying conditions. As the samples were anonymized, associations between hMPV infection and specific underlying diseases could not be assessed in this study.

Samples were sent to the Influenza Surveillance Network Laboratory for analysis within 48 h. We used commercially available Fluorescence Quantification PCR Kits (Jiangsu Bioperfectus Technologies Co., Jiangsu, China) to detect a range of viruses, including Influenza A virus H1N1, Influenza A virus H3N2, Influenza B virus (IBV), Adenovirus (ADV), RSV-A/B, hMPV, Human rhinovirus (HRV), and Parainfluenza virus types 1–4 (PIV-1/2/3/4). SARS-CoV-2 was detected using the 2019-nCoV Nucleic Acid Detection Fluorescent PCR Kit (JC10223-1N, Jiangsu Bioperfectus Technologies Co.). Co-infection was defined as detection of hMPV together with at least one additional respiratory virus including H1N1, H3N2, IBV, ADV, RSV-A/B, HRV and PIV-1/2/3/4.

### Whole-genome amplification and sequencing

The viral RNA was extracted using the QIAamp Viral RNA Mini Kit (QIAGEN, Hilden, Germany). The full-length genome was amplified using the hMPV A/B whole-genome enrichment kit (multiplex amplification method) (Micro-test, China). Whole-genome amplification and library preparation were performed according to the manufacturer’s instructions. Among hMPV-positive samples, those with relatively high viral load, sufficient residual sample volume and acceptable RNA quality were prioritized for whole-genome sequencing. To improve representativeness, samples were selected across different years and epidemic periods rather than from a single time point. The amplified hMPV genomic DNA underwent end repair, barcoding and adapter ligation using the Oxford Nanopore Technologies (ONT) Barcoding Assisted End-Repair and Ligation Kit (Micro-test, China) and Native Barcoding Kit 24 V14 (Oxford Nanopore Technologies, UK). The prepared library was loaded onto an R10.4.1 flow cell (FLO-MIN114) and sequenced using the GridION X5 platform (Oxford Nanopore Technologies, UK). Nanopore reads were assembled using a reference-guided approach in CLC Genomics Workbench v24. Quality-filtered reads were mapped to a representative hMPV reference genome using the ‘Map Reads to Reference’ function, and consensus sequences were generated using the ‘Extract Consensus Sequence’ function. Low-coverage or unreliable regions were masked as ‘N’ rather than filled from the reference sequence. Only complete or near-complete genomes passing quality control were retained for downstream analyses, resulting in 26 genomes.

### Amino acid variability and phylogenetic analysis of Ningbo strains

Amino acid variability of the G, SH and F proteins among the 26 Ningbo isolates was quantified using Protein Variability Server (PVS, http://imed.med.ucm.es/PVS/, access date: 13 December 2025) [9] in consensus sequence mode. Shannon entropy (H) was used for accessing per-residue entropy profiles from multiple sequence alignments generated by Multiple Alignment using Fast Fourier Transform v7.313 (MAFFT v7.313). Shannon entropy was calculated as:


$$H=-\sum_{i=1}^{M}p_{i}\log_{2}p_{i}$$

where *pi* is the frequency of amino acid type i at a given position, *M* is the number of amino acid types. Typically, positions where H≥2.0 are considered highly variable, while those where H<1.0 are regarded as highly conserved [10]. Lineage-specific substitutions were defined as amino acid residues conserved within one lineage but not observed in other lineages. Lineage-specific substitutions in the *F* gene were mapped onto a monomer *F* protein (PDB No. 5WB0) [11] and visualized in PyMOL v3.1.3.1 educational use only (Schrödinger, LLC, New York, NY, USA).

For phylogenetic analysis, we used Nextclade v3.21.1 to rapidly assign genotypes using *nextstrain/hmpv/all-clades/NC_039199* as reference dataset [12]. To generate a more comprehensive phylogenetic framework, we retrieved 949 publicly available hMPV sequences annotated as ‘complete genome’ from NCBI GenBank (access date: 20 April 2026; Supplementary information 1). Sequences containing more than 2% ambiguous sites, including N bases and degenerate/IUPAC nucleotide codes, were excluded. Multiple sequence alignment was performed using MAFFT v7.526 [13], and poorly aligned regions were removed with trimAl to enhance analytical reliability [14]. The optimal nucleotide substitution model was determined using ModelFinder, and the maximum likelihood (ML) tree was inferred using IQ-TREE 2.2.0 [15] with 5,000 ultrafast bootstrap replicates for branch support. All analyses were performed within the PhyloSuite platform V1.2.3 [16]. Strain AY640317.1 (Avian pneumovirus) was used as outgroup. Final lineage classification was then confirmed based on the ML tree. Sequence similarity analysis was carried out using Megalign V7.0.0 (DNAstar, USA) with clustalW to assess overall pairwise sequence similarity. SimPlot v3.5.1 was additionally used to generate similarity plots based on a sliding-window approach, with a window size of 200 bp, step size of 20 bp, GapStrip turned on, the Kimura two-parameter model, and a transition/transversion ratio of 2.0 [17]. Lineages of hMPV were designated according to previously reported nomenclature [4]. Neighbour-joining (NJ) trees of hMPV *F, SH* and *G* amino acid sequences were constructed in mega v11.0.13 with 1,000 bootstrap replicates to visually assess whether sequences clustered consistently with the lineage grouping inferred from the whole-genome ML phylogeny.

### Phylogeographic analysis of hMPV in Ningbo

To reconstruct the phylogeographic transmission dynamics of hMPV in Ningbo, we selected the *F*, *SH* and *G* genes for analysis. The aligned sequences were concatenated into a single dataset (*n*=174) for subsequent analysis.

Prior to phylogeographic analysis, temporal signal in the concatenated *F-SH-G* dataset was assessed using TempEst v1.5.3 by regressing root-to-tip genetic divergence against sampling time [18]. Phylogeographic inference was conducted using BEAST 2.7 [19], with location designated as a discrete trait to model geographic spread. The Best Model Test package was applied to determine the optimal substitution model, while an optimized Relaxed Clock model was chosen to account for rate variation among lineages. The Coalescent Skygrid Model was implemented as the tree prior to allow for flexible demographic reconstruction. Markov Chain Monte Carlo sampling was run for 1×10^8^ iterations to ensure robust estimation of evolutionary parameters. Convergence and effective sampling size (ESS) were assessed using Tracer v1.7.2 [20], with ESS>200 considered indicative of sufficient sampling and reliable parameter estimation. To summarize the phylogeographic results, Maximum Clade Credibility (MCC) trees were summarized in TreeAnnotator v2.7 after 10% burn-in, using median node heights. SPREAD v1.0.7 was then utilized to generate viral transmission pathways and evaluate Bayes Factor (BF), providing statistical support for different transmission routes [21]. Finally, Google Earth pro 7.3.7.1155 (Google LLC, Mountain View, CA, USA) was used to visualize the geographic spread of hMPV, allowing for an intuitive representation of viral movement across different regions. BF support was interpreted as follows: 3≤BF<10: medium support; 10≤BF<30: strong support; 30≤BF<100: very strong support and BF ≥100: extremely strong support.

### Statistical analysis

To evaluate the differences in hMPV-positive rates by year, season and age group, we employed the chi-squared test. This test was used to determine whether the distribution of positive rates significantly differed among various categories. For hMPV, each epidemic season was defined from the first week with ≥2 consecutive weeks of ≥3% positivity to the last such week, even when the season spanned 2 calendar years [22]. To evaluate temporal trends in co-infection, the Cochran–Armitage test for trend was used to assess whether the proportion of co-infections among hMPV-positive cases changed significantly across the ordered study years (2020–2024). All *P*-values were two-sided, and statistical significance was set at *P*<0.05.

## Results

### Temporal patterns of hMPV circulation in Ningbo

**Fig. 1.**
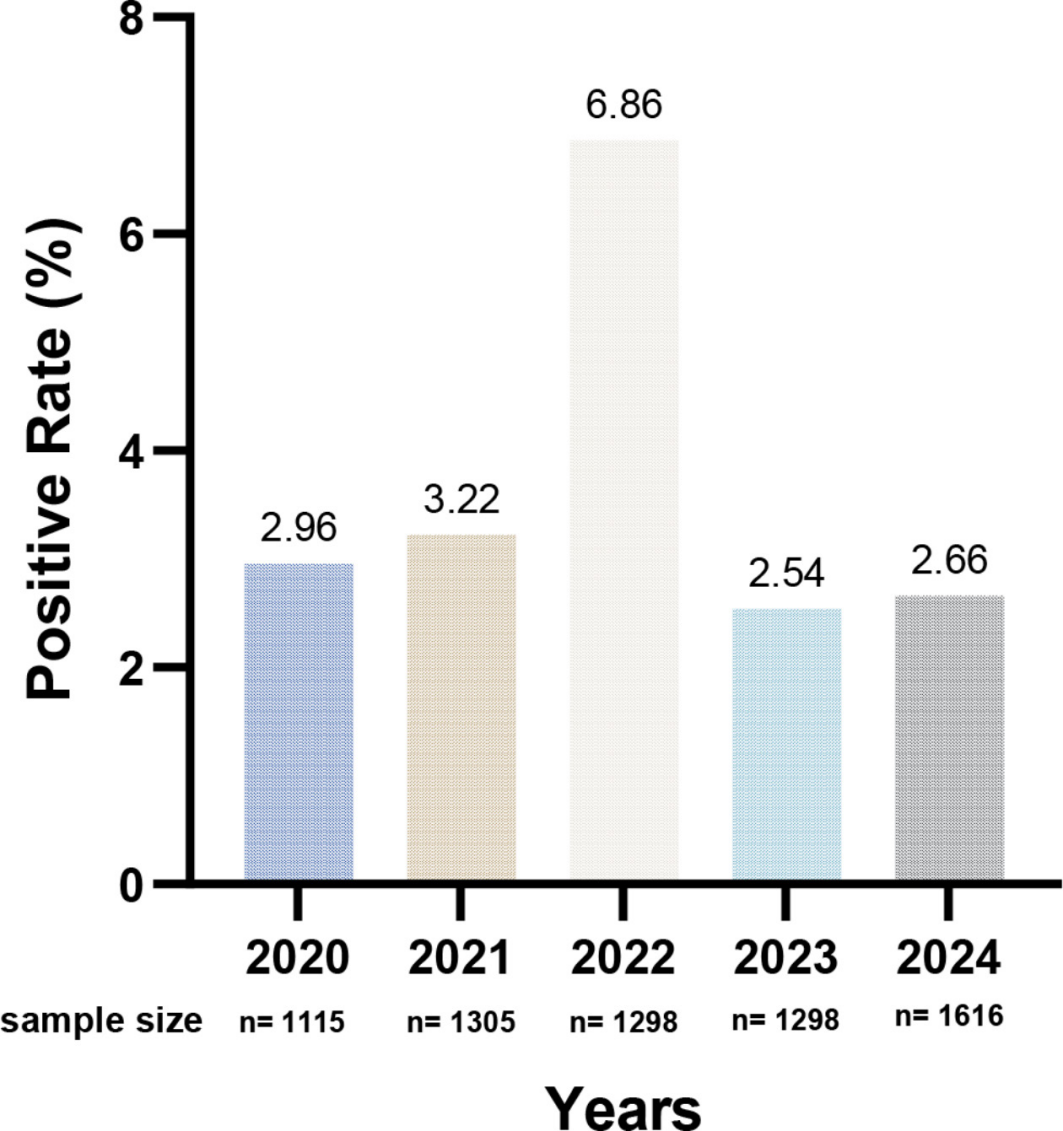
Annual positive rate of hMPV in Ningbo. The positive rate in 2022 was significantly higher than in other years (*P*<0.001).

To investigate the epidemiological characteristics of hMPV infections in children, we collected and analysed 6,632 clinical samples from 2020 to 2024. The overall hMPV-positive rate was 3.62% (240/6,632) (Fig. 1). Chi-square analysis revealed significant year-to-year variation in positive rates (χ²=49.6, *P*<0.001), with notably higher rates observed in 2022 compared to other years.

**Fig. 2.**
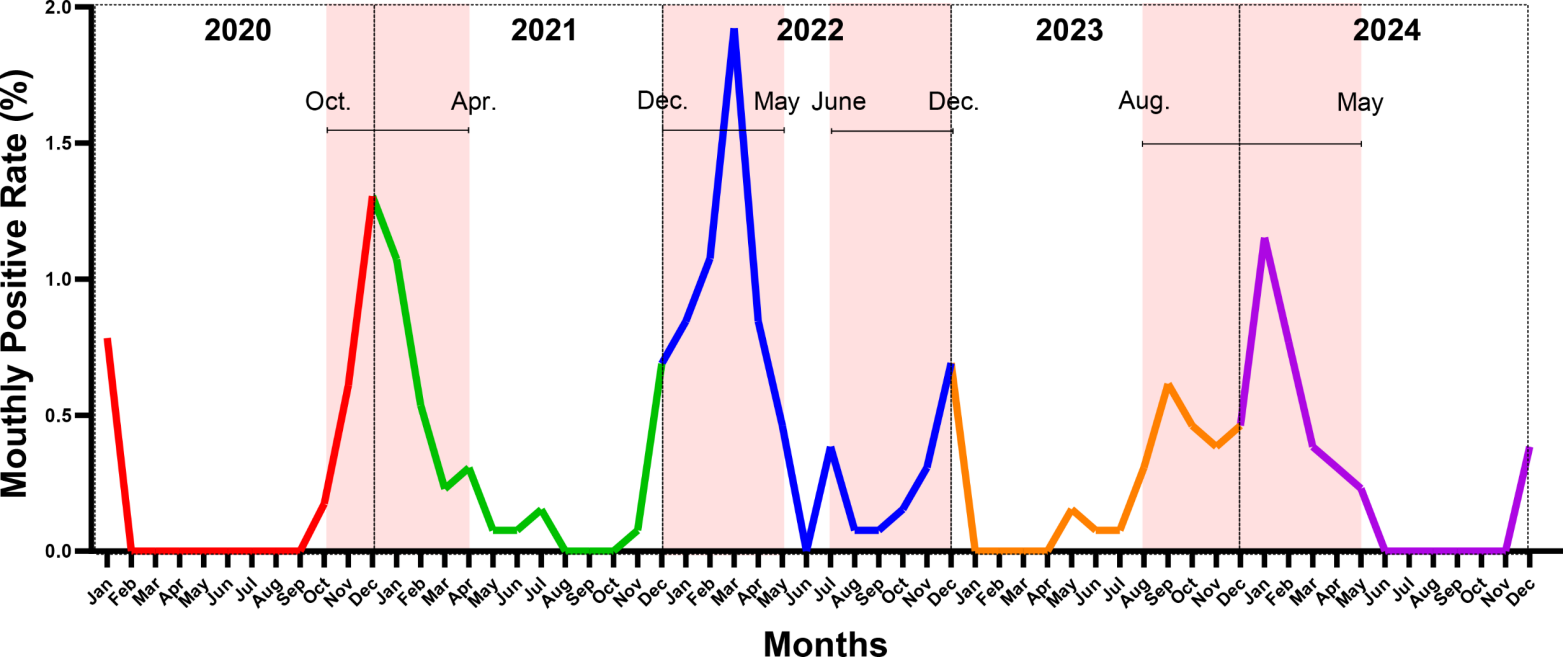
Monthly positive rate of hMPV in Ningbo. The epidemic season was highlighted in pink. The y-axis represents the monthly positive rate (%) of hMPV. Epidemic season was defined using weekly positivity data, from the first week with at least two consecutive weeks of weekly positivity ≥3% to the last such week, even when the season spanned 2 calendar years.

In seasonal distribution, the hMPV activity in Ningbo generally began in autumn, peaked in winter or the following spring, and subsided during the summer. The highest positive rate was observed in winter (December, January and February; 2.16%), which was significantly higher than the rates observed in spring (March, April and May; 1.30%), summer (June, July and August; 0.59%) and autumn (September, October and November; 0.50%) (χ²=104.91, *P*<0.001) (Fig. 2). Based on the predefined threshold of ≥3% weekly positivity for at least 2 consecutive weeks, four major hMPV epidemic seasons were identified during the study period. The 2020–2021 season showed main activity from October 2020 to April 2021, with sporadic detections extending to July 2021. The 2021–2022 season occurred between December 2021 and May 2022. The 2022–2023 season was confined to July–December 2022. The most recent 2023–2024 season began in August 2023 and persisted until May 2024, with only sporadic cases detected between May and July 2023 (Fig. 2).

### Age and co-infection characteristics of hMPV

We further analysed the demographic and co-infection characteristics of hMPV-positive children. Among the 240 hMPV-positive cases, 190 (79.2%) occurred in children aged 1–6 years, whereas only 7 (2.9%) occurred in infants aged <1 year and 6 (2.5%) in children aged >12 years (Table 1), indicating that hMPV-positive cases were predominantly observed in preschool-aged children.

**Table 1.** Demographic, seasonal, age distribution and co-infection characteristics of hMPV-positive children, 2020–2024

Characteristic	Overall	**2020**	**2021**	**2022**	**2023**	**2024**
No. of positive patients for hMPV	240	33	42	89	33	43
Male, n (%)	117 (48.8)	17 (51.5)	19 (45.2)	48 (53.9)	12 (36.4)	21 (48.8)
Female, n (%)	123 (51.2)	16 (48.5)	23 (54.8)	41 (46.1)	21 (63.6)	22 (51.2)
Age, median (IQR), years	4.0 (2.0–5.0)	4.0 (3.0–5.0)	4.0 (2.0–5.8)	3.0 (3.0–5.0)	4.0 (3.0–6.0)	3.0 (2.0–5.5)
<1, n (%)	7 (2.9)	1 (3.0)	1 (2.4)	5 (5.6)	0 (0.0)	0 (0.0)
1–6, n (%)	190 (79.2)	27 (81.8)	33 (78.6)	71 (79.8)	25 (75.8)	34 (79.1)
7–12, n (%)	37 (15.4)	5 (15.2)	7 (16.7)	12 (13.5)	8 (24.2)	5 (11.6)
>12, n (%)	6 (2.5)	0 (0.0)	1 (2.4)	1 (1.1)	0 (0.0)	4 (9.3)
Winter, n (%)	125 (52.1)	24 (72.7)	30 (71.4)	34 (38.2)	6 (18.2)	31 (72.1)
Spring, n (%)	64 (26.7)	0 (0.0)	8 (19.0)	42 (47.2)	2 (6.1)	12 (27.9)
Summer, n (%)	15 (6.2)	0 (0.0)	3 (7.1)	6 (6.7)	6 (18.2)	0 (0.0)
Autumn, n (%)	36 (15.0)	9 (27.3)	1 (2.4)	7 (7.9)	19 (57.6)	0 (0.0)
Co-infection, n (%)	41 (17.1)	2 (6.1)	4 (9.5)	13 (14.6)	7 (21.2)	14 (32.6)

Percentages are column percentages within each year. Age groups were defined as <1, 1–6, 7–12 and >12 years. Age is presented as median (IQR), with quartiles calculated using midpoint method. Seasons were defined as winter (December–February), spring (March–May), summer (June–August) and autumn (September–November). Co-infection was defined as detection of hMPV with at least one additional respiratory virus.

The proportion of co-infections among hMPV-positive patients showed a significant increasing trend over time (Cochran–Armitage trend test, χ²=12.00, two-sided, *P*<0.001), increasing from 6.1% (2/33) in 2020 to 32.6% (14/43) in 2024 (Table 1 and Supplementary information 2). The co-infecting viruses included RSV, IBV, ADV, HRV, PIV-1/3/4, H1N1 and H3N2, with IBV and RSV being the most common co-infecting viruses (Supplementary information 2). No co-infection of hMPV with SARS-CoV-2 was detected during the study period.

### Phylogenetic analysis of hMPV Ningbo strains

**Fig. 3.**
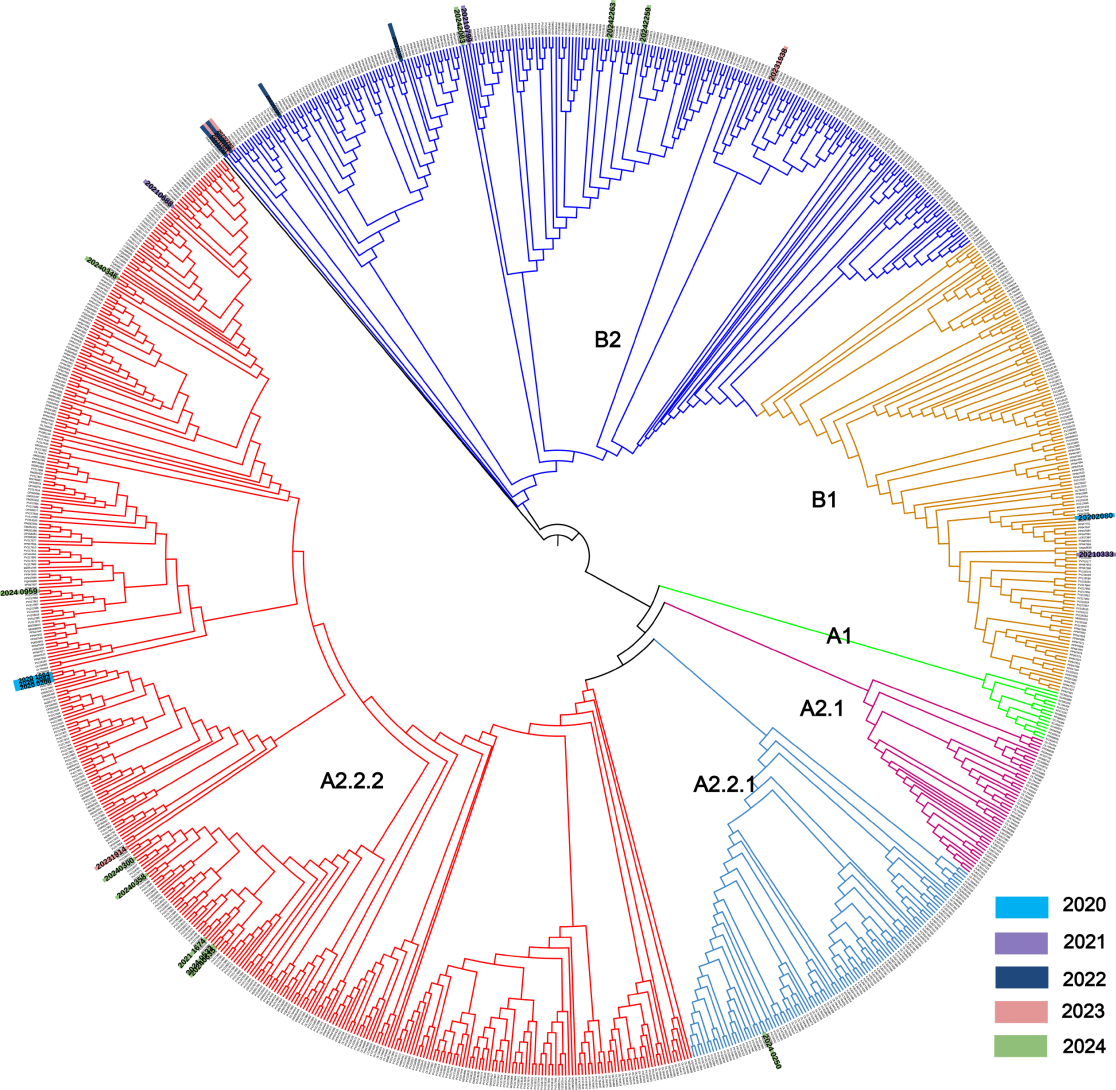
Phylogenetic analysis of hMPVs. (**a**) Complete genome (nucleotide) ML tree. The ML tree was constructed using 949 complete hMPV genomes from NCBI. MAFFT and trimAl were used for alignment, and ModelFinder and IQ-TREE in PhyloSuite for nucleotide model selection and ML tree inference. Different lineages were highlighted by different branch colours. Strain AY640317.1 (Avian pneumovirus) was used as outgroup. The 26 strains from 2020 to 2024 isolated in this study were highlighted by bars with different colour. Branch length was ignored. (**b**) NJ tree of amino acids for the *F*, *SH* and *G* genes of Ningbo strains.

We amplified the whole-genomes of 26 strains from positive samples collected from 2020 to 2024. Nextclade assignment showed that these 26 Ningbo strains belonged to four clades: A2.2.1 (*n*=1), A2.2.2 (*n*=12), B1 (*n*=2) and B2 (*n*=11). ML phylogenetic analysis supported Nextclade assignment (Fig. 3a). Among the sequenced strains, both A and B group viruses were detected across multiple years, with A2.2.2 and B2 being the most frequently observed lineages in this sequenced subset. B1 strains were detected in 2020 and 2021, whereas the single A2.2.1 strain was detected in 2024. NJ trees inferred from the amino acid sequences of the F, SH and G proteins also showed clear separation of hMPV sequences into the major genetic groups A and B (Fig. 3b), consistent with the whole-genome ML phylogeny.

### Molecular diversity and amino acid variability

**Fig. 4.**
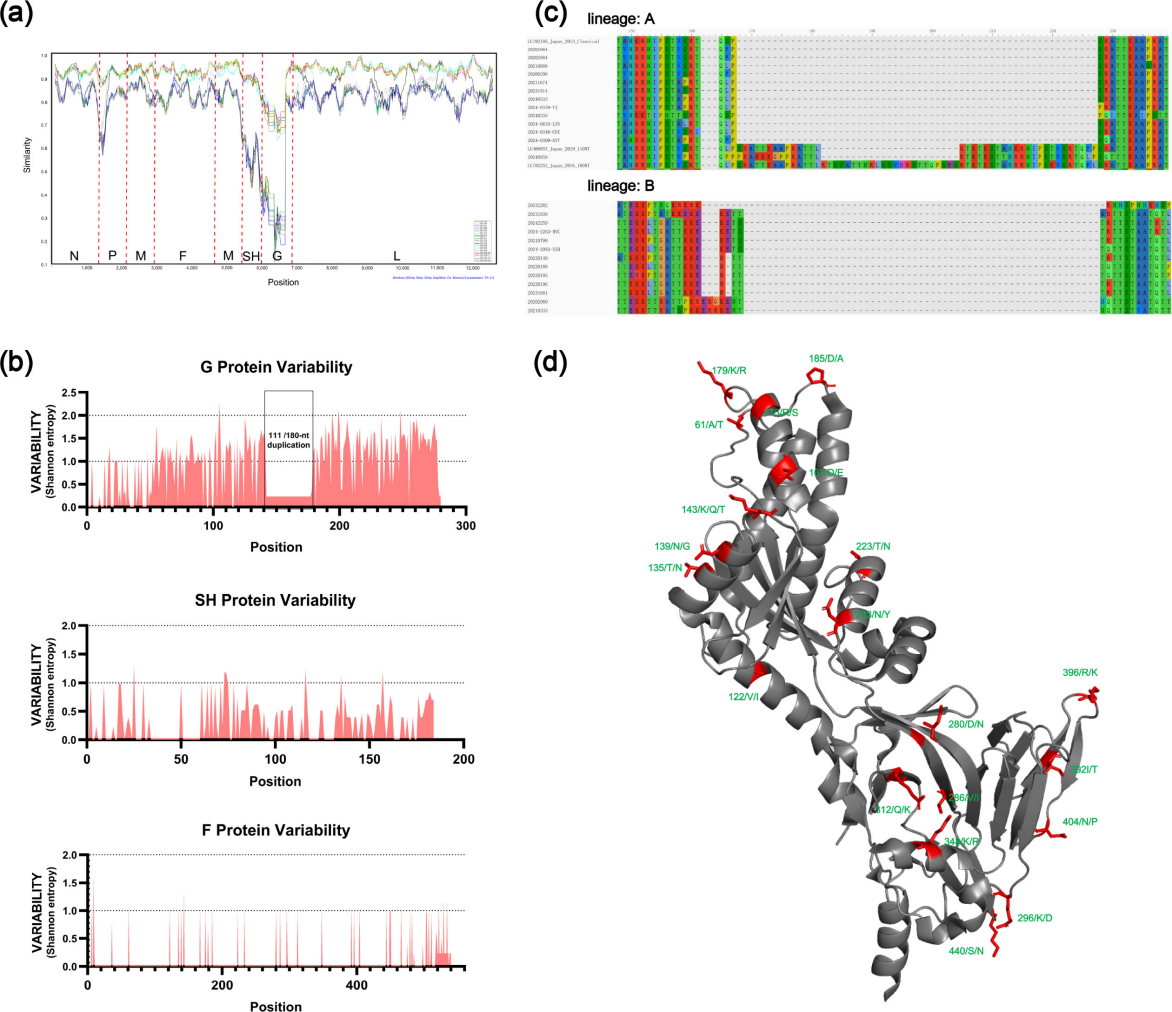
Molecular diversity and amino acid variability of Ningbo strains. (**a**) Sliding-window similarity plot of Ningbo hMPV genomes versus representative prototype strains. Nucleotide sequence similarity between the 26 Ningbo hMPV isolates and a reference strain (GenBank: NC_039199.1) was analysed using a sliding window approach (window size: 200 bp; step size: 20 bp) with the Kimura two-parameter model. Major coding regions were labelled for genomic context. (**b**) Shannon entropy (h) analysis between Ningbo strains. Positions where H≥2.0 are considered highly variable, while those where H<1.0 are regarded as highly conserved. (**c**) Amino acid alignment of the hMPV G-protein duplication region in Ningbo and reference strains. Amino acid sequences were aligned using MAFFT. The A2.2.2 strain (20240959) carrying G-protein duplication region was collected from 2024. (**d**) Mapping of lineage-associated substitutions on the F-protein monomer (PDB No. 5WB0). Lineage-associated substitutions are highlighted in red.

Sequence alignment of the 26 Ningbo near-complete genomes (individual genome lengths: 13,081–13,392 nt) showed that pairwise nucleotide sequence similarity ranged from 81.0 to 99.8%. Further analysis using Simplot revealed that the *G* (52.1–99.8%) and *SH* (54.6–99.8%) genes exhibited higher heterogeneity compared to the reference strains (Fig. 4a), consistent with previous reports [23]. The SimPlot curves further showed low sequence similarity in the *G* and *SH* regions and a clear separation into two clusters at the whole-genome level representing lineages A and B, consistent with the phylogenetic tree. Shannon entropy profiling revealed that the F protein was relatively conserved, with most residues showing H<1.0 and no sites exceeding H=2.0. The SH protein showed moderate variability, with a small number of residues reaching H>1.0 but none above H=2.0. In contrast, the G protein displayed the highest heterogeneity, with numerous sites having H>1.0 and several sites exceeding H=2.0 (Fig. 4b).

Previous studies have described three *G* gene length variants associated with partial duplications: a classical form without duplication and variants carrying 111-nt (37-aa) or 180-nt (60-aa) insertions [24]. We therefore examined whether similar duplication patterns were present in the Ningbo strains. We found that one A2.2.2 strain (20240959) collected from 2024 contained a 111-nt (37-aa) insertion in the *G* gene, whereas the other A2.2.2 strains showed the classical non-duplicated *G* sequence. In addition, several B-lineage strains exhibited distinct length polymorphisms in the same region when compared with the classical sequence: two B1 strains (20202080 and 20210333) had a three-amino acid insertion immediately upstream of the putative duplication site; one B2 strain (20232282) had a four-amino acid deletion in this region and seven B2 strains carried a single amino acid insertion within this region (Fig. 4c). Per-residue substitution analysis did not identify stable lineage-defining substitutions in *G* or *SH* across the lineages present. In the F protein, several lineage-associated substitutions were observed between lineages A, B1 and B2. When we mapped them onto the protein structure of *F*, we found four lineage-associated positions, 404 (N/P), 348 (K/R), 312 (Q/K) and 179 (K/R), are located within previously defined antigenic sites on the hMPV F protein [25] (Supplementary information 3, Fig. 4d).

### Phylogeographic analysis of hMPV in Ningbo based on the *F*-*SH*-*G* genes

**Fig. 5.**
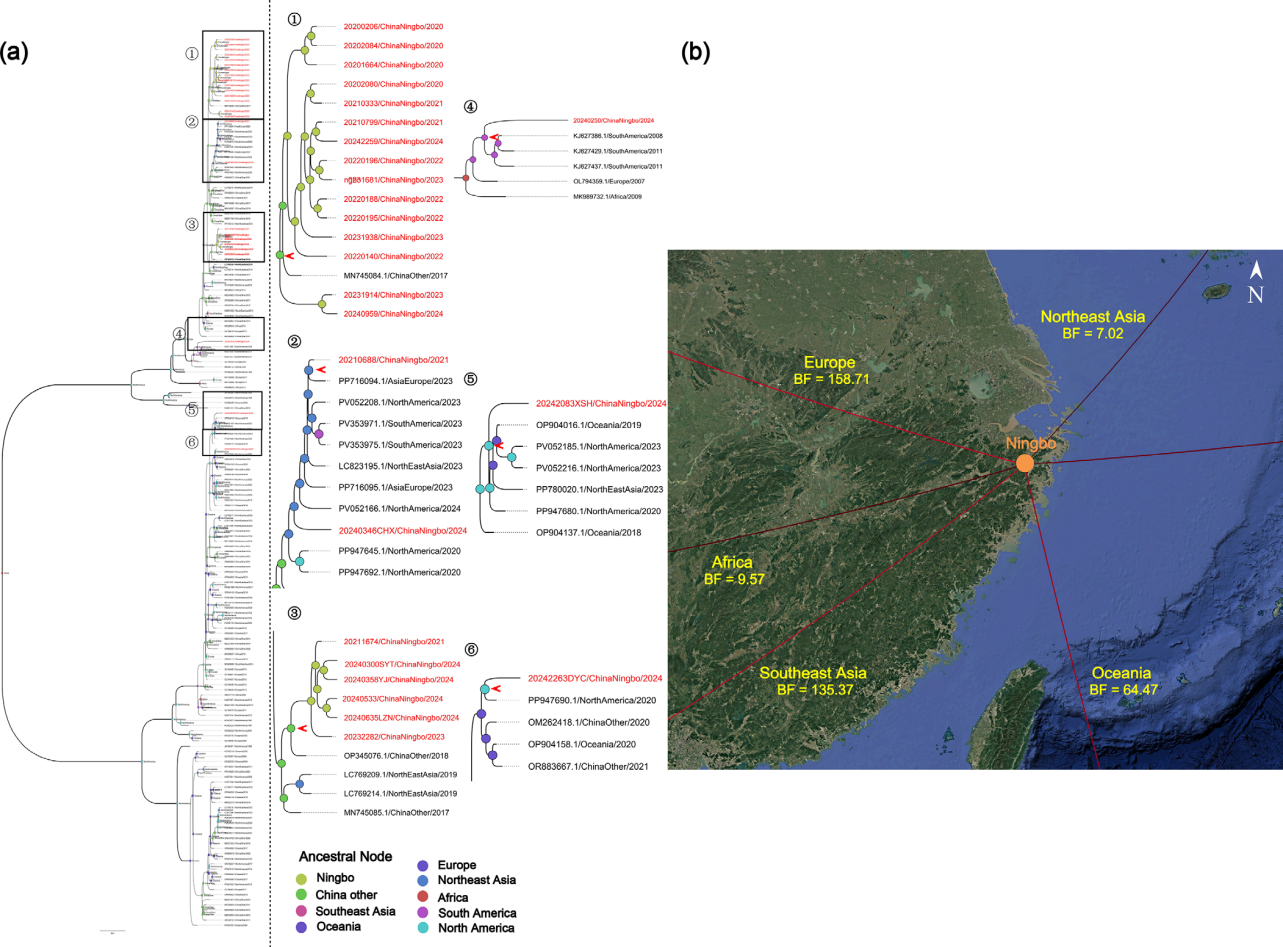
Phylogeographic analysis of hMPV in Ningbo based on the concatenated *F*, *SH* and *G* genes (*n*=174). (**a**) Discrete MCC Tree. Enlarged views of the evolutionary branches related to the Ningbo strains are shown in panels ① to ⑥. Ningbo strains are highlighted in red font, and ancestral nodes are indicated by red arrows. (**b**) Geographic transmission pathways based on Discrete BF. A composite dataset of 174 sequences was analysed using BEAST 2.7, with geographic locations designated as discrete traits. BF was used to assess statistical support for transmission routes, with BF≥3 indicating statistically significant support.

To investigate the transmission dynamics of hMPV in Ningbo, we reconstructed its global geographic dissemination using *F*, *SH* and *G* genes (*n*=174). Root-to-tip regression analysis showed a positive relationship between sampling time and genetic divergence (R²=0.7714), suggesting temporal structure in the dataset and supporting its use for exploratory molecular clock analysis (Supplementary information 5). We employed both the Discrete Tree and Discrete BF approaches to comprehensively assess the transmission patterns of hMPV across different geographic regions. In the Discrete Tree analysis, the MCC tree provided exploratory evidence consistent with possible cross-regional viral exchange and multiple introductions of hMPV into Ningbo from both domestic and international regions. Based on the 26 Ningbo strains analysed in the study, domestic circulation appeared to represent the main potential exchange pathway. Internationally, the ancestral nodes suggested possible introductions from North America, Northeast Asia and South America (Fig. 5a). To further quantify the strength of viral transmission between locations, we applied the discrete BF approach, which evaluates the statistical support for individual diffusion routes. Pathways with BF >3 were considered statistically supported transmission routes[26]. We identified potential hMPV exchange pathways between Ningbo and several geographic regions, including Europe (BF=158.71), Southeast Asia (BF=135.37), Oceania (BF=64.47), North America (BF=15.84), Africa (BF=9.57) and Northeast Asia (BF=7.02).

## Discussion

First, we analysed the epidemiological characteristics of hMPV in Ningbo. We observed that, among children, the highest annual hMPV positivity during 2020–2024 occurred in 2022. At the seasonal level, a marked peak was seen in the spring of 2022, despite the continued implementation of stringent COVID-19 related non-pharmaceutical interventions in China at that time. In addition, hMPV prevalence among children varies regionally across China: Hangzhou (a neighbouring city of Ningbo, 7.14%, 2020–2021) [27], Nanjing (4.66%, 2021–2022) [28] and Beijing (1.6%, 2020–2024) [29]. These differences may reflect variation in climate, population density, healthcare utilization and testing practices between regions. In our cohort, preschool-aged children (1–6 years) were the most affected group, likely due to close contact and relatively lower immunity in daycare settings, consistent with previous reports [29,30].

Notably, the proportion of co-infections among hMPV-positive cases increased over time, from 6.1% in 2020 to 32.6% in 2024. Recent studies have shown that, after the COVID-19 pandemic, paediatric respiratory virus epidemiology shifted not only in seasonality and age distribution but also in co-infection patterns [2]. A study conducted in northern China showed that rate of co-infection with respiratory pathogens rose from 25.1% in 2022 to 45.1% in 2023 [31]. Another study also indicates that the rate of respiratory virus co-infection among children rose gradually between 2021 and 2023, peaking in mid-2023 [32]. Most co-infections occurred in children aged 1–6 years, with no clear sex predominance. Although co-infected cases extended into older age groups in 2024, the small number of cases aged >12 years is unlikely to explain the marked increase in co-infection. Instead, the higher co-infection rate may reflect broader post-pandemic co-circulation of multiple respiratory viruses. The relaxation of strict non-pharmaceutical interventions, such as mask wearing, school closures, social distancing, travel restrictions and enhanced hand hygiene, may be one possible explanation for the increase in respiratory infections and co-infections among children. During the COVID-19 period, lockdowns and other social control measures likely suppressed the transmission of many respiratory viruses. As these measures were gradually lifted, children experienced renewed exposure in high-contact settings such as schools and kindergartens, which may have facilitated the resurgence and co-circulation of respiratory viruses. This may also partly explain why preschool-aged children had the highest infection rate in our study. The immune debt hypothesis may provide one possible explanation for this phenomenon, although it should be interpreted cautiously.

In our study, hMPV activity in Ningbo generally began in autumn, peaked in winter or the following spring, and subsided during the summer. Compared with RSV, recent studies suggest that RSV generally has an earlier seasonal onset and a longer epidemic duration than hMPV. Although the two viruses often co-circulate during the broader cold-season respiratory virus period, hMPV activity usually occurs later and is only partially synchronized with RSV peaks. After the COVID-19 pandemic, both viruses showed disrupted seasonality; however, RSV more frequently exhibited off-season or prolonged outbreaks, whereas hMPV tended to retain a spring-skewed pattern or showed shifted/multiple peaks [2]. Consistent with these observations, we identified two major epidemic periods in Ningbo in 2022, one early and one late in the year. This suggests that the onset of hMPV circulation in late 2021 was delayed, while the main epidemic in the second half of 2022 shifted forward to July, resulting in off-season summer activity. In addition, the 2023–2024 epidemic season in Ningbo was markedly prolonged, lasting ~9 months, whereas the other seasons persisted for only ~5–6 months. Similar atypical seasonal patterns have been reported in various regions worldwide, including Western Australia [33], the Netherlands [34] and Spain [35], and may be influenced by non-pharmaceutical interventions during the COVID-19 period as well as climatic and precipitation anomalies [1,33, 36].

Our phylogenetic analysis showed that four lineages (A2.2.1, A2.2.2, B1 and B2) co-circulated in Ningbo between 2020 and 2024, with A2.2.2 (formerly A2b2) and B2 predominating across several seasons. Importantly, the Ningbo A2.2.2 and B2 genomes did not form a single tight local cluster; instead, they were interspersed among contemporaneous sequences sampled from other parts of China and from overseas in the global background tree, consistent with repeated introductions rather than long-term evolution from one local source. Globally, group A and group B viruses usually co-circulate within the same season and the relative predominance of A versus B shifts over time [29]. Several studies have shown that the A2.2.2 sublineage is undergoing sustained expansion and has become a dominant lineage in many regions [4,7]. A recent survey from Ningxia in northern China reported that A2.2.2 accounted for 92.31% of hMPV detections between October 2023 and September 2024 [8]. In addition to A2.2.2, the B2 lineage also represented a large proportion of circulating strains in Ningbo, particularly in 2022 and 2023. By contrast, in neighbouring Hangzhou, A2.2.2 remained overwhelmingly dominant between February 2022 and January 2023 [37].

At the gene level, the *G* gene showed the lowest sequence identity among all coding regions, in agreement with previous reports that *G* is the most variable hMPV gene [1]. Among A2.2.x strains, we observed both classical non-duplicated G sequences and variants with a 111-nt partial duplication. As described in previous studies, *G*-gene partial duplications appear to be restricted to A2.2.x lineages and have become a characteristic feature of this group [4,7, 24, 29, 38]. Partial duplications of 111- or 180-nt in the *G* gene have arisen independently and spread widely, becoming the predominant hMPV A variants in some seasons, which suggests that these viruses may have a selective advantage [4,7, 38]. Notably, despite the limited sample size in this study, we identified one A2.2.2 strain (20240959) carrying the characteristic 111-nt duplication. Variants with 111-nt or 180-nt partial duplications in the G gene have been increasingly reported in multiple regions, including Asia and Europe, and have become widespread in A2-related lineages [7,24, 39]. Its detection in Ningbo further supports the city’s status as a major international port, where frequent cross-border travel may facilitate the introduction of emerging viral genotypes from the global viral pool. In RSV, duplications in the *G* gene have also been described and are hypothesized to contribute to immune evasion by the virus [40,43]. However, although G-duplication variants are frequently detected among patients with severe acute respiratory infection and have become dominant in many regions [39], their precise impact on viral transmissibility and clinical severity remains unclear, and definitive functional evidence is still lacking.

Although the hMPV *F* gene was overall more conserved than *G* and *SH* in our Ningbo dataset (no highly variable sites with H≥2), the F protein remains the primary target of neutralizing antibodies and therefore is still epidemiologically meaningful to monitor at the residue level. Structurally, hMPV *F* is a trimeric class I fusion glycoprotein that adopts a metastable prefusion conformation and undergoes major refolding during membrane fusion, and multiple antigenic regions on *F* have been defined by structural studies: conserved neutralizing epitopes such as the DS7-binding site and antigenic sites shared/analogous to RSV *F*, including sites III/IV/V [11,44]. Recent human monoclonal antibody profiling further indicates that potent neutralizing activity against hMPV is frequently directed to diverse epitopes on the side of the F trimer, not only to a single ‘apex-dominant’ region [25,44, 45]. We observed several lineage-associated substitutions that differentiate lineages A, B1 and B2, which are also located on the lateral surface of the F trimer. Some substitutions are of potential interest because some coincide with previously defined antigenic sites on the hMPV F protein [25]. However, no functional or serological data were available in the present study, and therefore their effect on antigenicity or antibody recognition remains unknown.

Phylogenetic analysis showed that the Ningbo isolates are closely related to strains from both China and other countries, suggesting that hMPV in Ningbo has likely been introduced multiple times from different sources. In our time-scaled phylogenetic tree, Ningbo A2.2.2 and B2 strains were interspersed among contemporary sequences from other parts of China and from overseas rather than forming a single local cluster, indicating that these viruses are part of internationally distributed post-pandemic clades. The Discrete Tree analysis provided exploratory evidence consistent with multiple introductions of hMPV into Ningbo from both domestic and international regions. Based on the 26 Ningbo strains analysed, domestic circulation appeared to represent the main potential exchange pathway, while ancestral-node reconstruction also suggested possible introductions from overseas regions. In the Discrete BF analysis, potential hMPV exchange pathways were observed between Ningbo and several geographic regions, including Europe, Southeast Asia, Oceania, North America, Africa and Northeast Asia. However, these phylogeographic results should be interpreted cautiously. BEAST-based discrete phylogeographic inference is sensitive to the number and geographic distribution of available sequences. Because public hMPV genomic data are unevenly distributed across regions, and because only 26 Ningbo genomes were available in this study, the inferred ancestral locations and BF-supported pathways may be affected by sampling density and regional underrepresentation. Therefore, we interpret these results as exploratory evidence supporting possible cross-regional connectivity and multiple introductions into Ningbo, rather than as definitive evidence of direct transmission from any specific country or region. Despite these limitations, the overall pattern is consistent with Ningbo’s role as a highly connected coastal port city, where frequent domestic and international movement may facilitate the introduction and circulation of diverse hMPV lineages. Ningbo is home to the Ningbo–Zhoushan Port located in the east of China, the world’s largest port by cargo throughput, which operates over 300 international container shipping routes and connects with more than 600 ports in nearly 200 countries and regions across all five continents, including major markets in Europe, North America, South America, Oceania and Asia. Global shipping hubs like Ningbo facilitate high levels of human mobility and logistical exchange, which may promote the introduction and spread of respiratory viruses, as observed during the COVID-19 pandemic [46,47]. Similar patterns have been observed in other port cities, where increased connectivity has been associated with the emergence and circulation of various infectious diseases [48].

This study has several limitations. First, our surveillance and sequencing data were available only for the period from 2020 to 2024, and no pre–COVID-19 hMPV data from the same setting were included. As a result, the observed year-to-year differences in positive rates, seasonal patterns and circulating lineages reflect only the pandemic and post-pandemic period and cannot be directly compared with a true pre-pandemic baseline. Because of data and ethical constraints, we did not collect detailed clinical information such as hospitalization or hypoxia. Therefore, we could not formally compare disease severity between mono-infections and co-infections. Second, because genome recovery depended on sample quality, viral load and specimen availability, the sequenced 26 genomes represent a quality-filtered, temporally distributed subset rather than a random sample of all hMPV-positive cases. Additionally, all samples collected in a single outpatient fever clinic in Ningbo may not be fully generalizable to all paediatric populations in the city or in other regions. Third, our phylogeographic analysis was based on publicly available sequences from other regions. Because some regions had limited or no sequence data, they were underrepresented. This may have introduced uncertainty into the inferred transmission routes and should be considered when interpreting the results.

In conclusion, this study characterized the epidemiological trends and genetic diversity of hMPV in Ningbo from 2020 to 2024, highlighting notable seasonal variation and epidemic peaks in 2022. Preschool-aged children were the most affected group, and the co-infection rate reached 34.88% in 2024. Genetic analysis identified two main co-circulating sublineages (A2.2.2 and B2). Phylogeographic reconstruction revealed viral exchanges between Ningbo and multiple domestic and international regions. This study represents the multi-year investigation of hMPV in Ningbo, uncovering cross-regional viral movement in a globally connected context. While some findings are partially consistent with previous reports, this work provides temporal and geographic insights into hMPV evolution and spread.

## Supplementary material

10.1099/mgen.0.001760 Supplementary Material 1.

10.1099/mgen.0.001760 Supplementary Material 2.

10.1099/mgen.0.001760 Supplementary Material 3.

10.1099/mgen.0.001760 Supplementary Material 4.

## Abbreviations

ADV: adenovirus
BF: Bayes Factor
ESS: effective sampling size
hMPV: human metapneumovirus
HRV: human rhinovirus
IBV: influenza B virus
MCC: Maximum Clade Credibility
ML: maximum likelihood
NJ: neighbour joining
PIV: parainfluenza virus
PVS: Protein Variability Server
RSV: respiratory syncytial virus
