# Supplementary material for: Epidemiological characterization and phylogenetic analysis of human metapneumovirus isolated from children in Ningbo, China, 2020–2024
Source: Microb Genom. 2026 Jun 22;12(6):001760. doi: 10.1099/mgen.0.001760 (PMC13286607; doi:10.1099/mgen.0.001760)
Supplement: Supplementary Material 4. [file mgen-12-01760-s004.pdf]

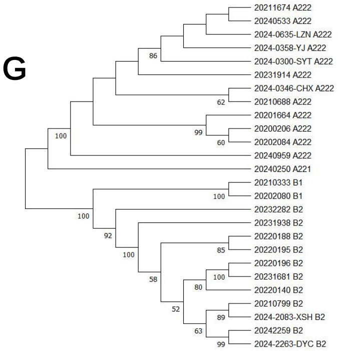

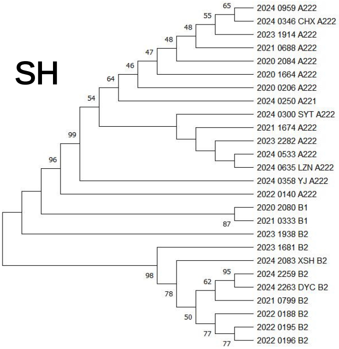

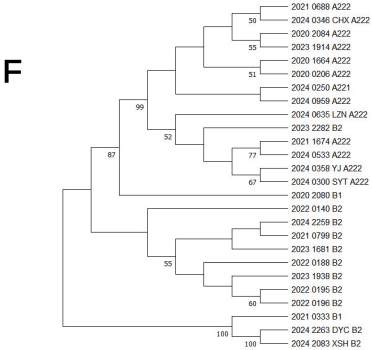

Supplementary information 4

Neighbor-Joining tree of amino acids for the *F*, *SH* and *G* genes from Ningbo strains.

Date range	42
Slope (rate)	1.1997E-3
X-Intercept (TMRCA)	1752.9779
Correlation Coefficient	0.8783
R squared	0.7714
Residual Mean Squared	2.0589E-5

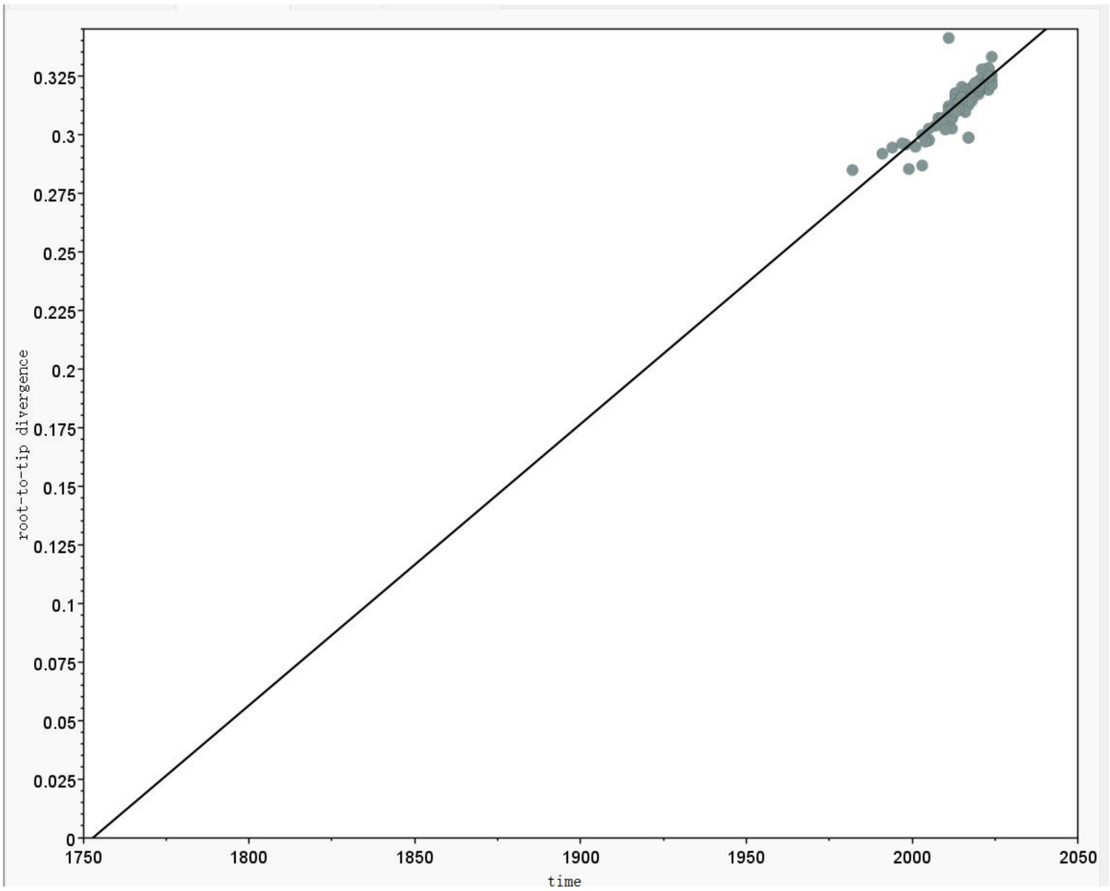

Supplementary Figure 5 Root-to-tip regression plot
